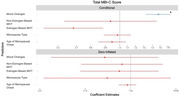# Cognitive and behavioral decline predicted by perimenopausal symptoms: A CAN‐PROTECT study

**DOI:** 10.1002/alz.092052

**Published:** 2025-01-03

**Authors:** Jasper F. E. Crockford, Dylan X. Guan, Gillian Einstein, Eric E. Smith, Zahinoor Ismail

**Affiliations:** ^1^ University of Calgary, Calgary, AB Canada; ^2^ Hotchkiss Brain Institute, University of Calgary, Calgary, AB Canada; ^3^ University of Toronto, Toronto, ON Canada

## Abstract

**Background:**

Menopause has been associated with greater dementia risk. We investigated the relationship between the presence of specific symptoms experienced during menopause (i.e., perimenopausal symptoms) and later‐life emergence of cognitive and behavioral symptoms that are linked to elevated dementia risk.

**Method:**

Participant data were from the Canadian Platform for Research Online to Investigate Health, Quality of Life, Cognition, Behaviour, Function, and Caregiving in Aging (CAN‐PROTECT) study. The sample for this analysis comprised 896 post‐menopausal participants, who recalled the presence or absence of 11 perimenopausal symptoms. Symptoms were categorized as vasomotor, weight changes, vaginal dryness, irregular periods, sleep problems, mood changes, brain fog, and other unnamed symptoms. Currently experienced subjective cognitive symptoms were measured with the Everyday Cognition (ECog‐II) scale. Emergent and persistent neuropsychiatric symptoms were measured with the Mild Behavioral Impairment Checklist (MBI‐C). Higher scores reflected greater severity on both measures. A negative binomial regression examined the association between perimenopausal symptoms and cognitive function. A zero‐inflated negative binomial regression examined the association between perimenopausal symptoms and MBI symptoms. All models adjusted for age of menopausal onset, menopausal hormone therapy (MHT), menopause type (i.e., spontaneous, or due to medical reasons), age, and years of education.

**Result:**

Symptoms of brain fog, weight changes, and mood changes were associated with poorer current ECog‐II score (b = 74.8, 95%CI [47.2, 108.0], p<.001; b = 24.4, 95%CI [8.9, 42.2], p = .001; b = 36.2, 95%CI [17.3, 58.3], p<.001, respectively). Comparatively, use of estrogen and non‐estrogen‐based MHT during menopause was not significantly associated with current ECog‐II scores (b = ‐11.0,95%CI[‐25.3, 6.5], p = .2; b = 16.9, 95%CI[‐10.9, 56.1], p = .3, respectively). Weight and mood symptoms of perimenopause were significantly associated with poorer current MBI‐C score (b = 24.4,95%CI[2.4,51.1], p = .03; b = 68.4%,95%CI[36.3,108.1], p<.001, respectively). MBI‐C scores differed based on type of MHT used. Estrogen‐based MHT was associated with a statistically significant 26.9% lower MBI‐C score (95%CI[‐43.3, ‐5.7], p = .02), while score did not differ significantly with non‐estrogen‐based MHT (b = ‐19.1,95%CI[‐44.6, 18.1], p = .3).

**Conclusion:**

The experience of brain fog, weight changes, and mood changes during perimenopause may predict greater risk for cognitive and behavioral changes later on. Use of estrogen‐based MHT may mitigate the relationship between perimenopause symptoms and MBI symptoms. However, longitudinal data are required to explore mechanisms.